# Supplementary material for: Base-resolution UV footprinting by sequencing reveals distinctive damage signatures for DNA-binding proteins
Source: Nat Commun. 2023 May 11;14:2701. doi: 10.1038/s41467-023-38266-2 (PMC10175305; doi:10.1038/s41467-023-38266-2)
Supplement: Supplementary file 1 — Supplementary Information [file 41467_2023_38266_MOESM1_ESM.pdf]

Elliott et al.

Supplementary appendix

Table of Contents

Supplementary Figure 1 2
Supplementary Figure 2 3
Supplementary Figure 3 4
Supplementary Figure 4 5
Supplementary Figure 5 6
Supplementary Figure 6 7
Supplementary Figure 7 8
Supplementary Figure 8 9
Supplementary Figure 9 10
Supplementary Figure 10 11
Supplementary Figure 11 12
Supplementary Table 1 13
Supplementary Table 2 14
Supplementary Table 3 15
Supplementary references 16

Supplementary Figure 1

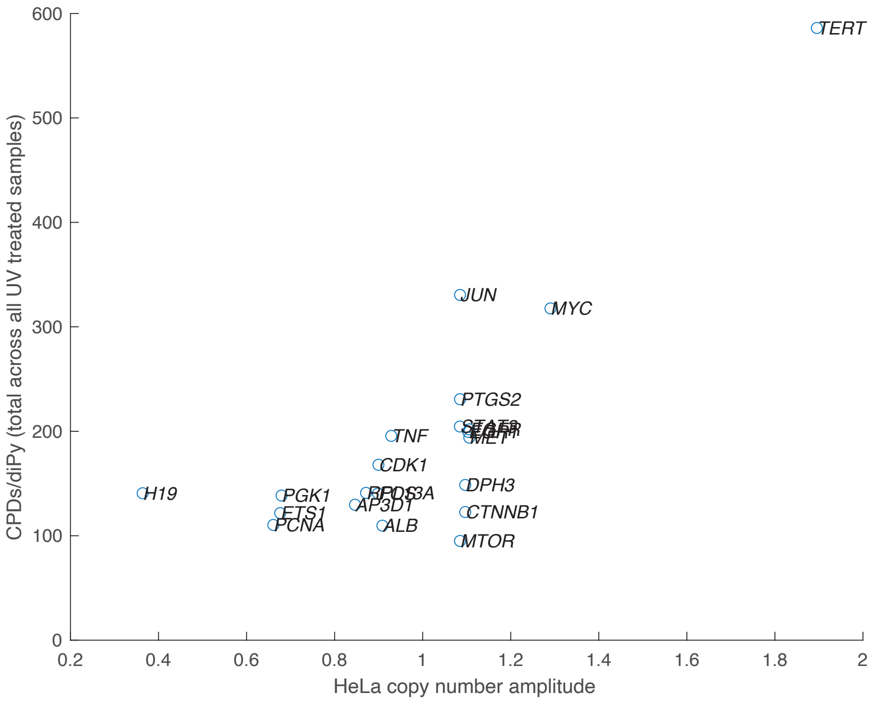

Per-region CPD coverage correlates with HeLa genomic copy number. Copy number amplitudes were derived from Cancer Cell Line Encyclopedia (CCLE) data. Source data are provided as a Source Data file.

Supplementary Figure 2

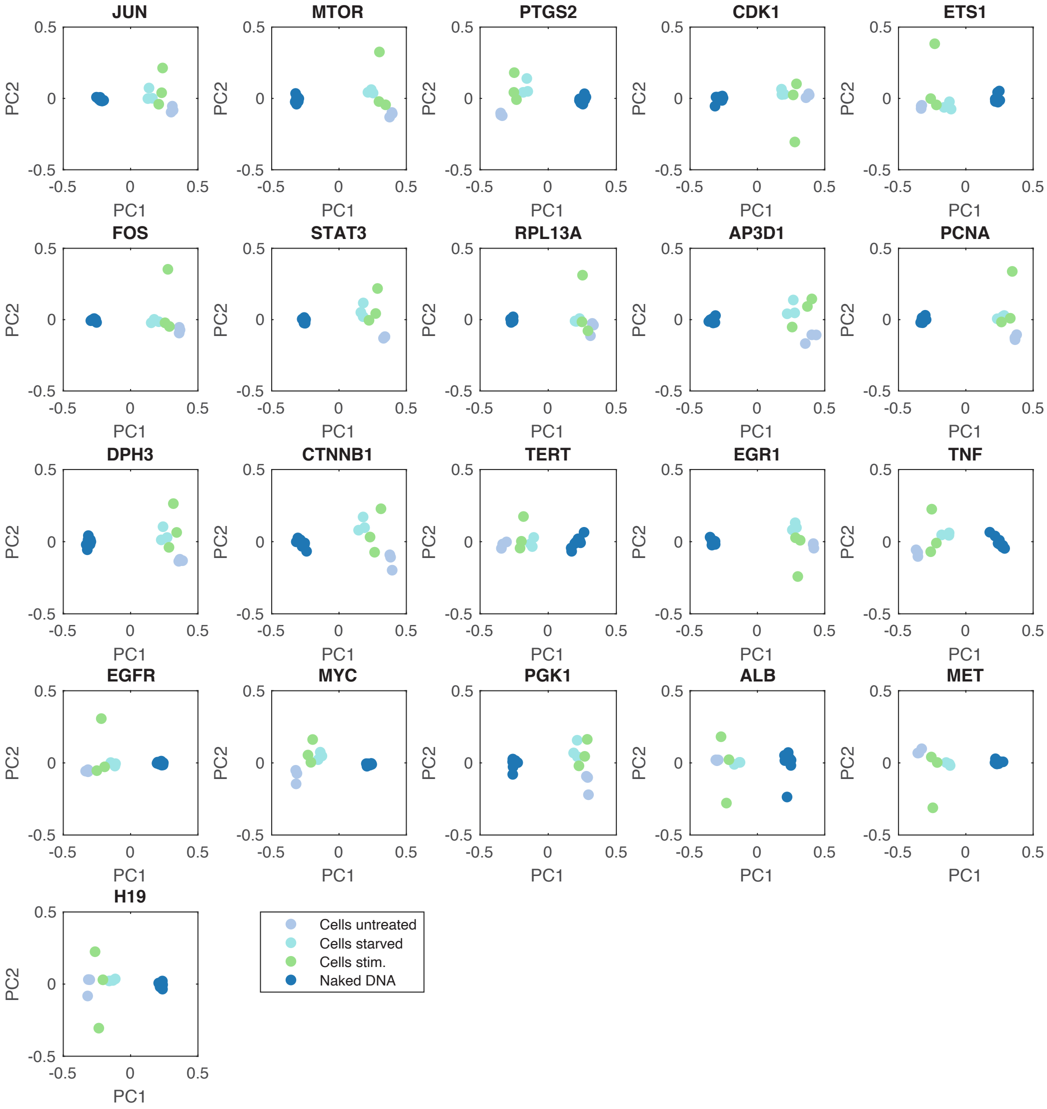

PCA analysis of quantitative CPD data. For each region, the CPD level vectors (3-5 kb in size) were analyzed using principal components analysis (PCA) to reveal similarities and dissimilarities between conditions and replicates. PC1, principal component 1; PC2, principal component 2. Source data are provided as a Source Data file.

Supplementary Figure 3

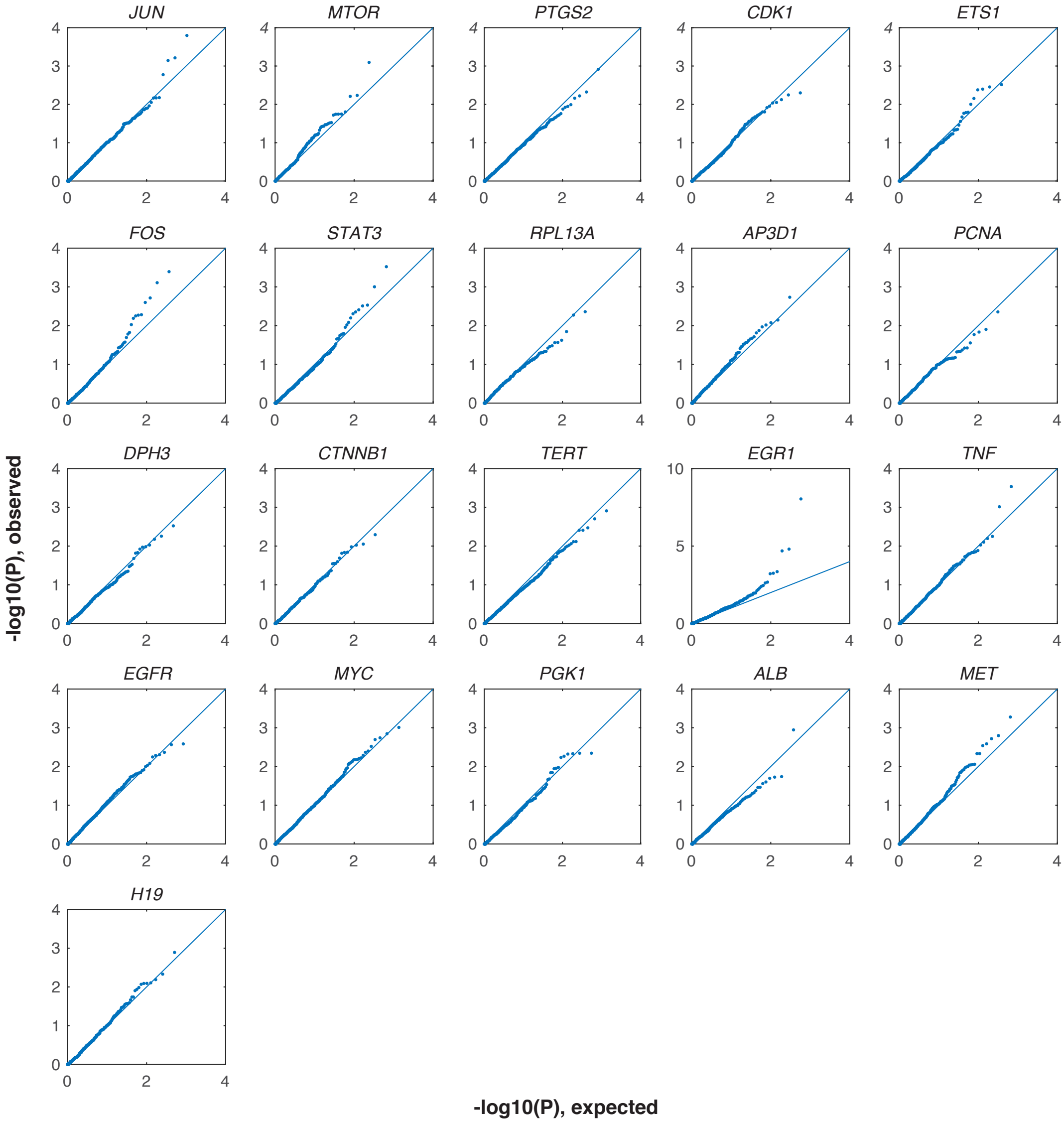

Statistical assessment of individual positions showing altered CPD formation in serum stimulated vs. starved conditions. The quantile-quantile plots describe expected (uniform) vs. observed *P*-values for individual positions in each region. An independent filter was used, such that only positions exhibiting at least 10 CPD detections on average across replicates were included. *P*-values were determined using a two-sided negative binomial test (see **Methods**). Source data are provided as a Source Data file.

Supplementary Figure 4

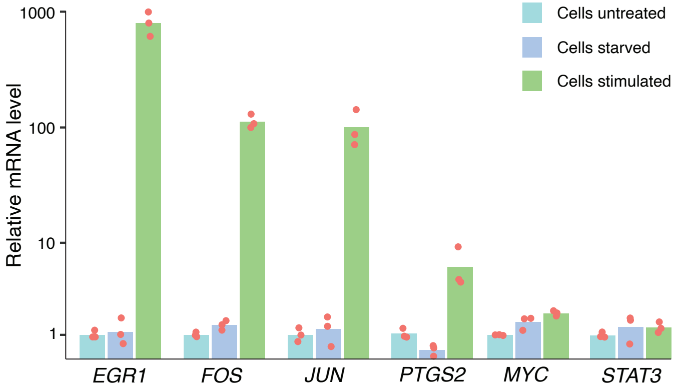

Confirmation of serum-responsiveness of EGR1, FOS, JUN and PTGS2 in HeLa using qPCR. mRNA levels, which were first normalized relative to an endogenous control (GAPDH), are shown relative to the untreated condition. MYC and STAT3 are also included as unresponsive controls. Barplot represents the mean and data points (red dots) are shown for three replicates. Source data are provided as a Source Data file.

Supplementary Figure 5

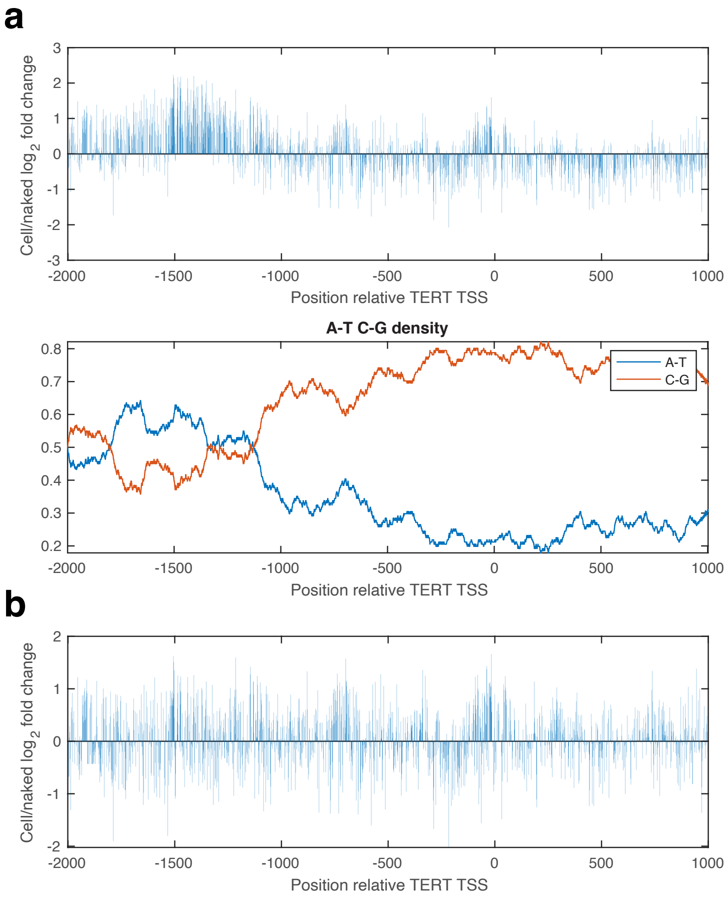

Local depth normalization corrects for GC-related biases. (**a**) Basic global per-region depth normalization of CPD profile data leads to local biases in cell vs. naked (acellular purified DNA) fold changes that correlate with sequence GC content (example from the *TERT* promoter). These regional differences in CPD formation frequency may be indicative of overall differences in basic physical/chemical CPD formation conditions in cellular compared to acellular samples, such as ion strength and nucleic acid concentration. (**b**) Local fold change bias in *TERT* corrected by using local depth normalization (250 bp windows). Source data are provided as a Source Data file.

Supplementary Figure 6

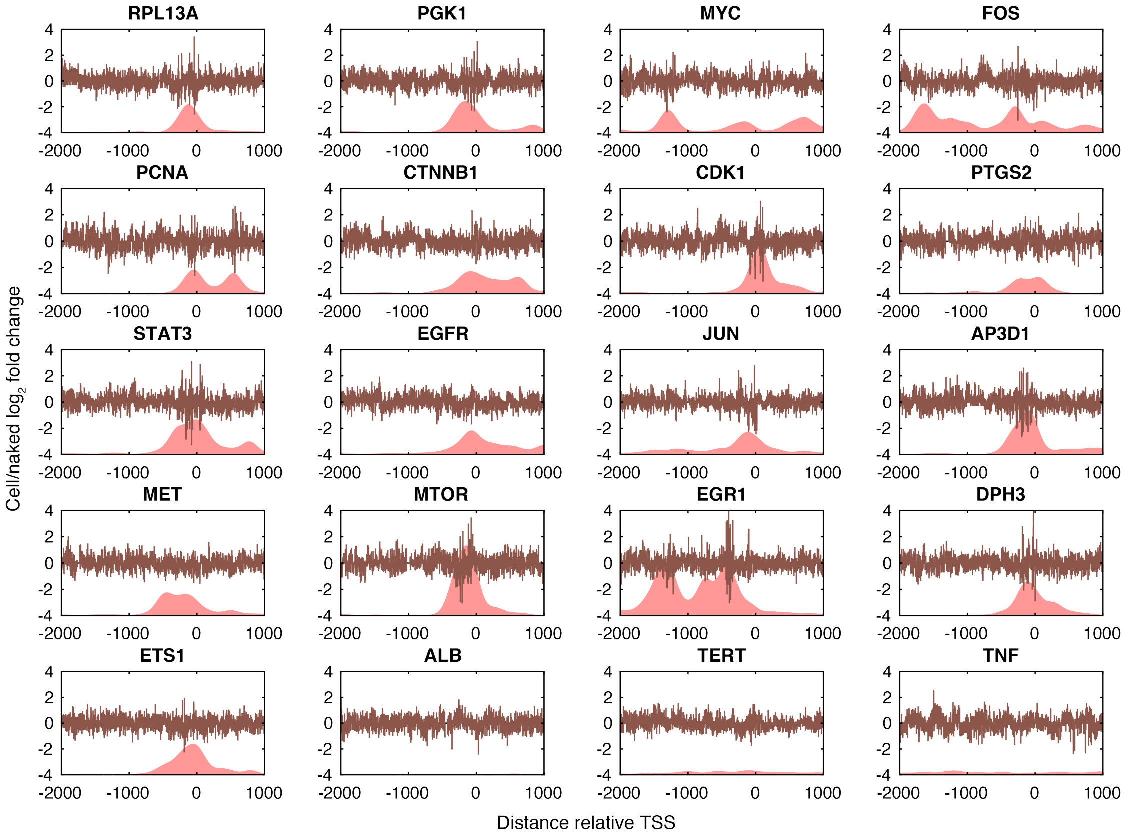

Per-base changes in CPD formation in cellular compared to naked samples. Differential CPD signals (brown) are plotted on a $\log_2$ scale, such that for example + 4 indicates a 16-fold increase in CPD formation at one specific base position. HeLa-S3 DNaseI hypersensitivity (DNaseI HS from ENCODE/OpenChrom, Duke University) is indicated for each region. Regions are ordered by expression level in HeLa-S3, with *RPL13A* having highest and *TNF* having lowest expression. Source data are provided as a Source Data file.

Supplementary Figure 7

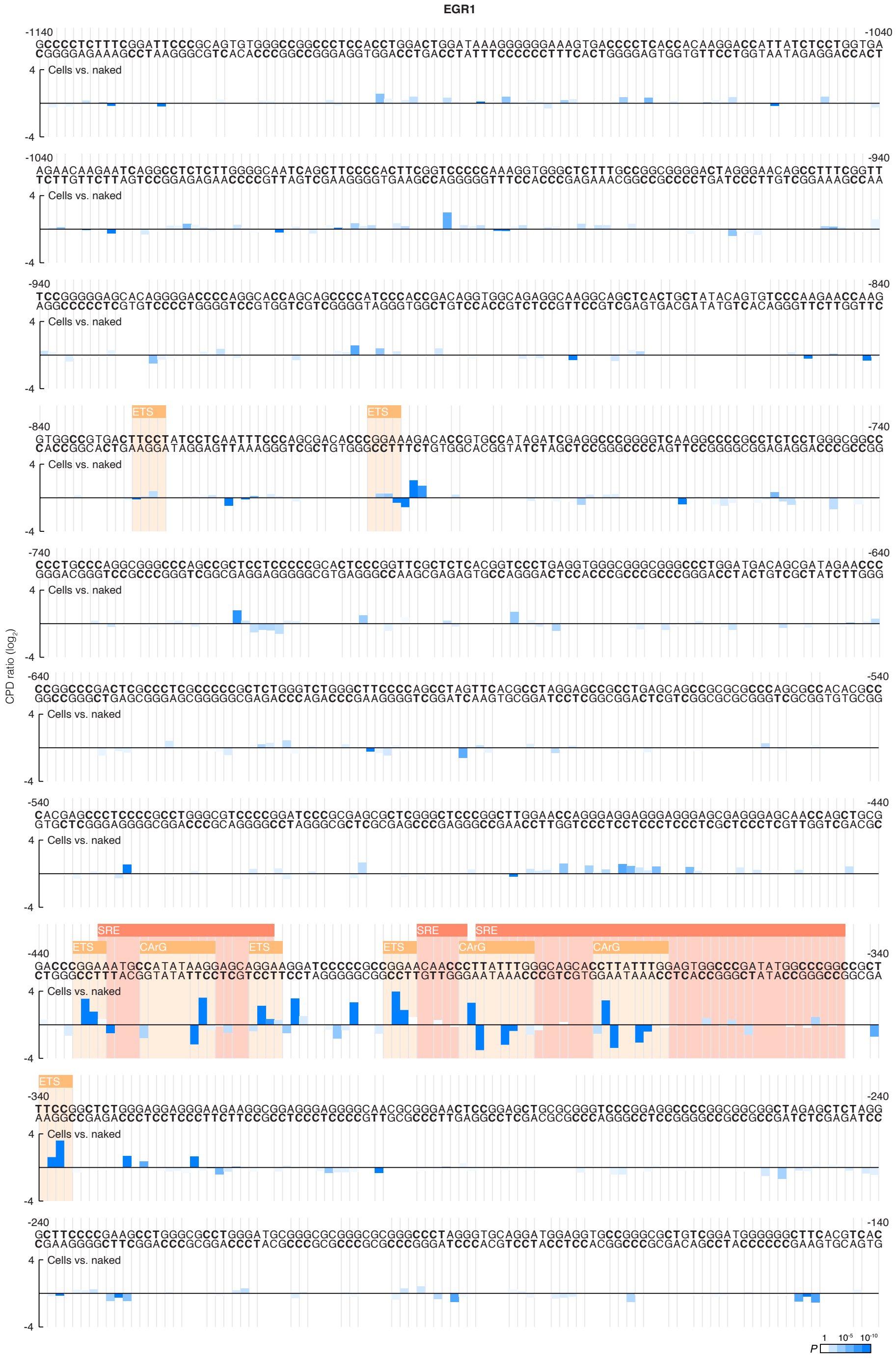

Base-resolution cell/naked CPD level ratios at -1140 to -140 bp relative to the TSS in *EGR1*. Known serum response elements (SREs) containing several predicted ETS and CArG sites are indicated, showing that CPD signals arise predominantly in these known regulatory elements. Bars are color-coded by significance at each base position. Source data are provided as a Source Data file.

Supplementary Figure 8

a

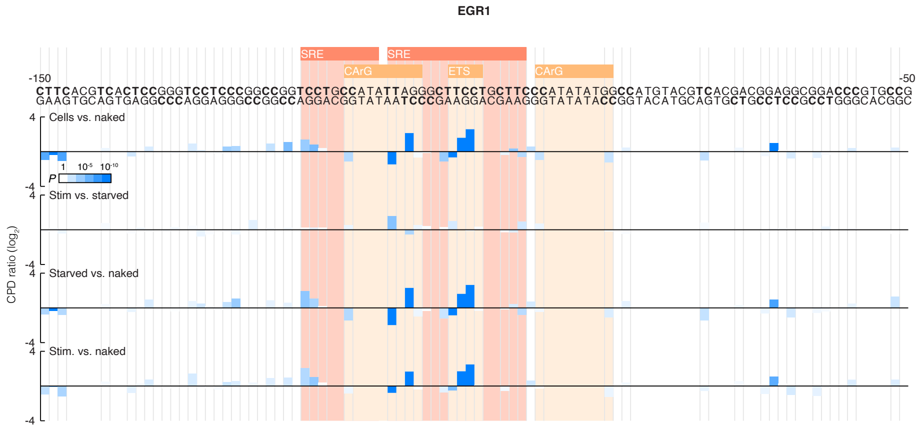

b

CArG CPD position	1	2	3	4	5	6	7	8	9
-115 bp EGR1 CArG box	C C	A	T	A	T	T	A	G	G
	G G	T	A	T	A	A	T	C	C
**Sample**									
Untreated 1	4	N/A	N/A	N/A	N/A	9	N/A	139	14
Untreated 2	4	N/A	N/A	N/A	N/A	7	N/A	82	4
Untreated 3	1	N/A	N/A	N/A	N/A	10	N/A	72	2
Starved 1	2	N/A	N/A	N/A	N/A	14	N/A	137	12
Starved 2	4	N/A	N/A	N/A	N/A	9	N/A	142	2
Starved 3	3	N/A	N/A	N/A	N/A	8	N/A	95	0
Stimulated 1	11	N/A	N/A	N/A	N/A	42	N/A	154	16
Stimulated 2	2	N/A	N/A	N/A	N/A	13	N/A	37	0
Stimulated 3	2	N/A	N/A	N/A	N/A	15	N/A	34	5
Naked 1	7	N/A	N/A	N/A	N/A	104	N/A	27	9
Naked 2	2	N/A	N/A	N/A	N/A	50	N/A	12	5
Naked 3	9	N/A	N/A	N/A	N/A	54	N/A	15	7
Naked 4	9	N/A	N/A	N/A	N/A	76	N/A	25	7
Naked 5	1	N/A	N/A	N/A	N/A	46	N/A	16	7
Naked 6	11	N/A	N/A	N/A	N/A	107	N/A	31	7
Naked 7	10	N/A	N/A	N/A	N/A	76	N/A	18	5
Naked 8	7	N/A	N/A	N/A	N/A	68	N/A	10	4
Naked 9	7	N/A	N/A	N/A	N/A	71	N/A	31	5

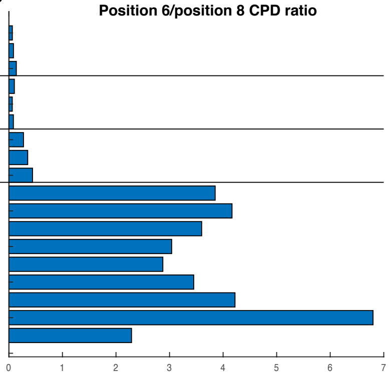

Subtle CPD changes at a CArG element in *EGR1* following serum stimulation. (**a**) Significant change in CPD formation (P = 2.06e-5, two-sided binomial test; q = 0.004 using Benjamini-Hochberg correction with independent filtering requiring 10 CPDs on average across conditions) in serum stimulated compared to starved samples was seen at -110 bp relative to the TSS in the *EGR1* promoter, coinciding with a CArG element starting at -115 bp. Comparison of stimulated vs. naked and starved vs. naked signals suggested a modest weakening of the CPD damage signature at this site upon serum stimulation. (**b**) Detailed investigation of the same signal change. Untreated and serum-starved cells exhibited sharp changes in CPD formation at this CArG element, involving stimulated damage at position 8 and inhibited damage at position 6 relative to naked DNA (the same signature was exhibited by a CArG in the *FOS* promoter, which also had informative dipyrimidines at the same positions; **Supplementary Fig. 9**). The CPD level ratio between these positions was thus highly skewed in these conditions compared to naked DNA. While the pos 6/pos 8 skew persisted in serum-stimulated replicates, it was also consistently reduced, thus suggesting a minor reduction in SRF occupancy. Source data are provided as a Source Data file.

Supplementary Figure 9

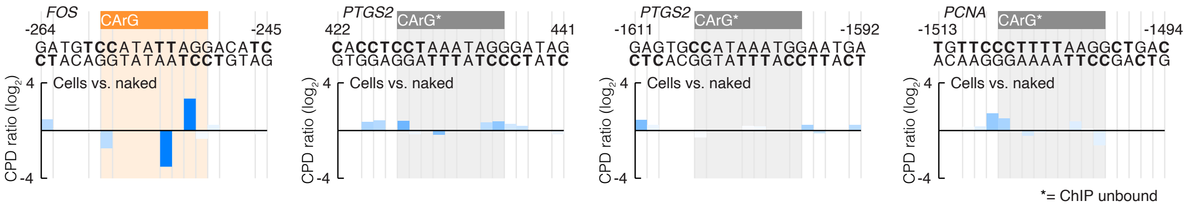

CPD damage patterns at additional CArG elements. Four additional CArGs were mapped in the targeted regions. Of these, one was bound by SRF according to ENCODE (GM12878) ChIP (*FOS*) while the remaining three lacked obvious ChIP support. The second PTGS2 site lacks a leading diPy. Source data are provided as a Source Data file.

Supplementary Figure 10

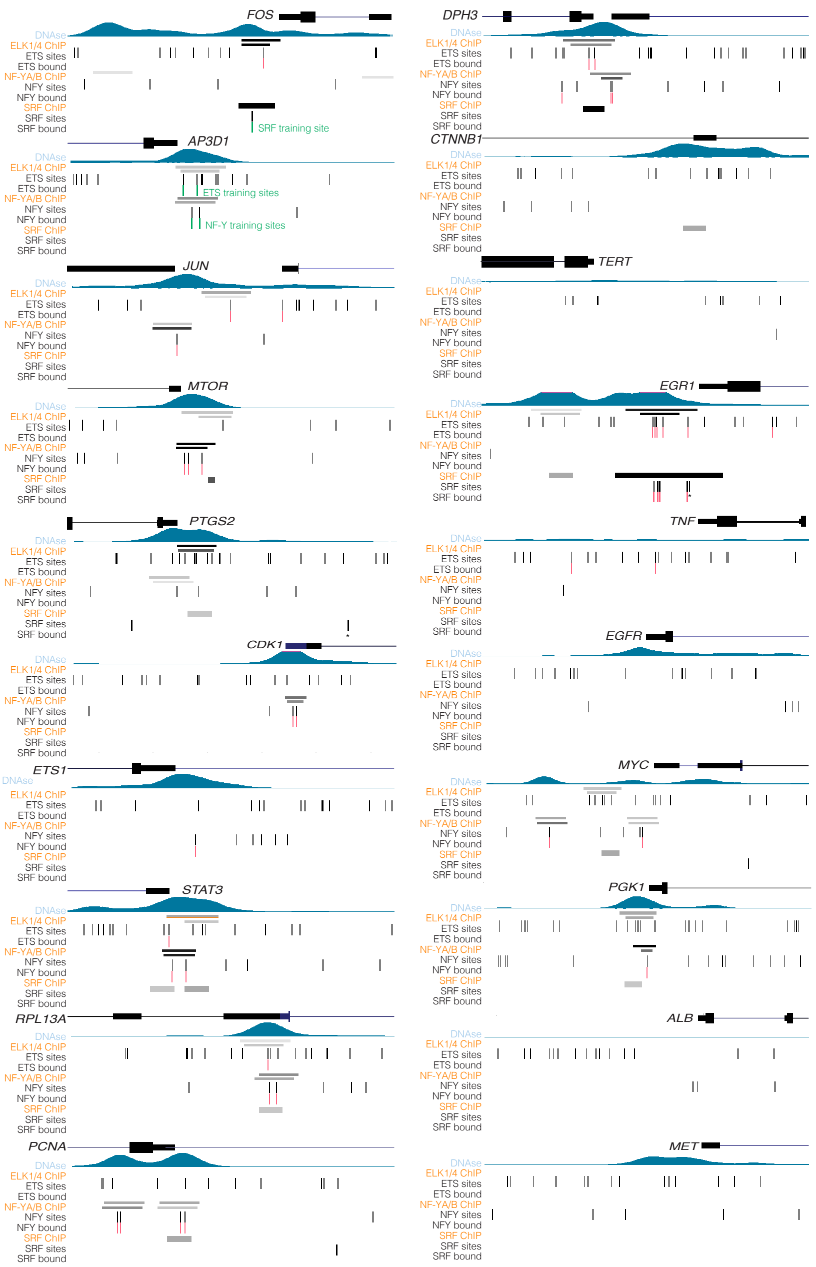

CPD-based prediction of SRF, ETS and NF-Y binding site occupancy. ChIP-supported motif-matching sites in *AP3D1* and *FOS* were used as positive training examples (indicated in green). Remaining candidate binding sites, identified based on sequence, were classified as bound (red lines) or unbound using a basic nearest neighbor classifier applied to cell/naked $\log_2$ CPD damage signals using Euclidian distance. A synthetic damage-neutral site (zero $\log_2$ ratios) were used as negative example. The following motifs and informative CPD positions (left-most position in each diPy is underscored) were used: SRF, CCT[A/T]$_5$GG; ETS, TTCC[G/T]; NF-Y, CCAAT. *, one CArG box in *EGR1* and one in *PTGS2* was not classified due to lacking the critical second (CT) diPy. ENCODE ChIP peak calls are included (darker gray means higher amplitude). Source data are provided as a Source Data file.

Supplementary Figure 11

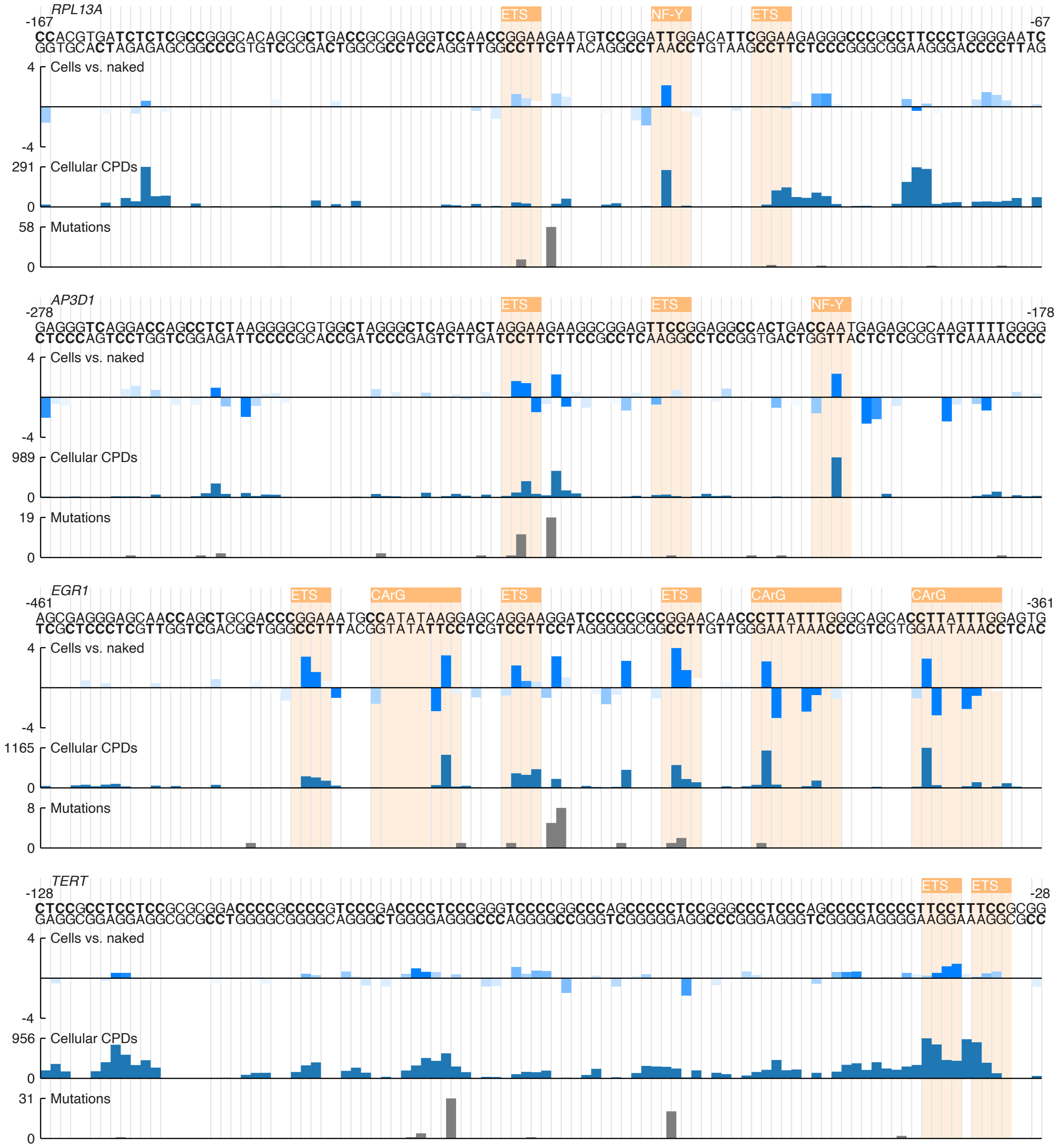

Melanoma somatic mutation hotspots vs. CPD damage patterns in additional promoters. In addition to *DPH3*, shown in main **Fig. 5d**, another four of the targeted regions harboured somatic mutation hotspots (>3 mutations) identified based on whole genome mutation calls from 221 melanomas (see **Methods**). Mutation counts at these sites (100 bp contexts) are shown together with per-base cell/naked CPD ratios and absolute CPD counts (cellular conditions). Source data are provided as a Source Data file.

Supplementary Table 1

Chromsome	Start	End	Gene	TSS pos	Size (bp)	Refs
chr1	59248785	59251785	*JUN*	59249785	3000	[1,2]
chr1	11321564	11324564	*MTOR*	11322564	3000	[3]
chr1	186648559	186651559	*PTGS2*	186649559	3000	[4]
chr10	62536088	62539088	*CDK1*	62538088	3000	[5,6]
chr11	128391205	128394205	*ETS1*	128392205	3000	[7]
chr14	75743476	75746476	*FOS*	75745476	3000	[2]
chr17	40539586	40542586	*STAT3*	40540586	3000	[8]
chr19	49988810	49991810	*RPL13A*	49990810	3000	[9-11]
chr19	2150565	2153565	*AP3D1*	2151565	3000	[9]
chr20	5099672	5102672	*PCNA*	5100672	3000	[2]
chr3	16305479	16308479	*DPH3*	16306479	3000	[9,10,12,13]
chr3	41238924	41241924	*CTNNB1*	41240924	3000	[14]
chr5	1294162	1297162	*TERT*	1295162	3000	[15,16]
chr5	137799178	137802178	*EGR1*	137801178	3000	[17]
chr6	31541343	31544343	*TNF*	31543343	3000	[18]
chr7	55084713	55088713	*EGFR*	55086713	4000	[19]
chr8	128745679	128749679	*MYC*	128747679	4000	[20]
chrX	77357670	77361670	*PGK1*	77359670	4000	[21]
chr4	74267955	74270955	*ALB*	74269955	3000	[22]
chr7	116310443	116313443	*MET*	116312443	3000	[23]
chr11	2019800	2024800	*H19*	*	5000	[24]

Regions assayed by Capture CPD-seq. Coordinates refer to the human hg19 assembly. Transcription start site (TSS) positions used in the study were derived from the GENCODE 19 annotation and generally based on the 5′-most coding transcript for each gene (some transcripts deemed irelevant in HeLa were disregarded). *, intergenic region near the *H19* long non-coding RNA. Refs indicate key references describing the promoters which were used to design the panel.

Supplementary Table 2

	Index	Sequence 5' to 3'
ARC49	(i5)<br>AGATCTCG	AATGATACGGCGACCAC**CGAGATCT**ACACTCTTTCCCTACACGACGCTCTTCCGATCT
ARC156	(i5)<br>TATAGCCT	AATGATACGGCGACCAC**AGGCTATA**ACACTCTTTCCCTACACGACGCTCTTCCGATCT
ARC157	(i5)<br>ATAGAGGC	AATGATACGGCGACCAC**GCCTCTAT**ACACTCTTTCCCTACACGACGCTCTTCCGATCT
ARC141/142		GTGACTGGAGTTCAGACGTGTGCTCTTCCGATCT*T<br>/5Phos/AGATCGGAAGAGCACACGTCTGAACTCCAGTCAC/3AmMO/
ARC143/144		/5Biosg/ACACTCTTTCCCTACACGACGCTCTTCCGATCTNNNNNN/3AmMO/<br>/5Phos/AGATCGGAAGAGCGTCGTGTAGGGAAAGAGTGT/3AmMO/
ARC154		ACACTCTTTCCCTACACGACGCTCTTCCGATCT
ARC78	(i7)<br>ATCACG	CAAGCAGAAGACGGCATACGAGAT**CGTGAT**GTGACTGGAGTTCAGACGTGTGCTCTTCCGATCT
ARC84	(i7)<br>CGATGT	CAAGCAGAAGACGGCATACGAGAT**ACATCG**GTGACTGGAGTTCAGACGTGTGCTCTTCCGATCT
ARC85	(i7)<br>TTAGGC	CAAGCAGAAGACGGCATACGAGAT**GCCTAA**GTGACTGGAGTTCAGACGTGTGCTCTTCCGATCT
ARC86	(i7)<br>TGACCA	CAAGCAGAAGACGGCATACGAGAT**TGGTCA**GTGACTGGAGTTCAGACGTGTGCTCTTCCGATCT
ARC87	(i7)<br>ACAGTG	CAAGCAGAAGACGGCATACGAGAT**CACTGT**GTGACTGGAGTTCAGACGTGTGCTCTTCCGATCT
ARC88	(i7)<br>GCCAAT	CAAGCAGAAGACGGCATACGAGAT**ATTGGC**GTGACTGGAGTTCAGACGTGTGCTCTTCCGATCT
ARC89	(i7)<br>CAGATC	CAAGCAGAAGACGGCATACGAGAT**GATCTG**GTGACTGGAGTTCAGACGTGTGCTCTTCCGATCT
ARC90	(i7)<br>ACTTGA	CAAGCAGAAGACGGCATACGAGAT**TCAAGT**GTGACTGGAGTTCAGACGTGTGCTCTTCCGATCT
ARC91	(i7)<br>GATCAG	CAAGCAGAAGACGGCATACGAGAT**CTGATC**GTGACTGGAGTTCAGACGTGTGCTCTTCCGATCT

Primers used in CPD-seq

Supplementary Table 3

gene	FH sequence	RH sequence	Amplicon size (bp)
EGR1	GCAGAGTCTTTTCCTGAC	TTGGTCATGCTCACTAGG	194
FOS	CAGTTATCTCCAGAAGAAGAAG	CTTCTAGTTGGTCTGTCTCC	130
JUN	AAAGGATAGTGCGATGTTTC	TAAAATCTGCCACCAATTCC	189
MYC	TGAGGAGGAACAAGAAGATG	ATCCAGACTCTGACCTTTTG	86
STAT3	GGTACATCATGGGCTTTATC	TTTGCTGCTTTCACTGAATC	98
PTGS2	AAGCAGGCTAATACTGATAGG	TGTTGAAAAGTAGTTCTGGG	113
MMP1	AAAGGGAATAAGTACTGGGC	CAGTGTTTTCCTCAGAAAGAG	130
GAPDH	CTTTTGCGTCGCCAG	TTGATGGCAACAATATCCAC	139

Kickstart primers (Sigma) used for qPCR of serum stimulation

Supplementary references

1 Rozek, D. & Pfeifer, G. P. In vivo protein-DNA interactions at the c-jun promoter in quiescent and serum-stimulated fibroblasts. *J Cell Biochem* **57**, 479-487, doi:10.1002/jcb.240570313 (1995).

2 Tornaletti, S. & Pfeifer, G. P. UV light as a footprinting agent: modulation of UV-induced DNA damage by transcription factors bound at the promoters of three human genes. *J Mol Biol* **249**, 714-728, doi:10.1006/jmbi.1995.0331 (1995).

3 Bendavit, G., Aboulkassim, T., Hilmi, K., Shah, S. & Batist, G. Nrf2 Transcription Factor Can Directly Regulate mTOR: LINKING CYTOPROTECTIVE GENE EXPRESSION TO A MAJOR METABOLIC REGULATOR THAT GENERATES REDOX ACTIVITY. *J Biol Chem* **291**, 25476-25488, doi:10.1074/jbc.M116.760249 (2016).

4 Grall, F. T. *et al.* The Ets transcription factor ESE-1 mediates induction of the COX-2 gene by LPS in monocytes. *FEBS J* **272**, 1676-1687, doi:10.1111/j.1742-4658.2005.04592.x (2005).

5 Badie, C., Itzhaki, J. E., Sullivan, M. J., Carpenter, A. J. & Porter, A. C. Repression of CDK1 and other genes with CDE and CHR promoter elements during DNA damage-induced G(2)/M arrest in human cells. *Mol Cell Biol* **20**, 2358-2366, doi:10.1128/MCB.20.7.2358-2366.2000 (2000).

6 Tommasi, S., Oxyzoglou, A. B. & Pfeifer, G. P. Cell cycle-independent removal of UV-induced pyrimidine dimers from the promoter and the transcription initiation domain of the human CDC2 gene. *Nucleic Acids Res* **28**, 3991-3998, doi:10.1093/nar/28.20.3991 (2000).

7 Majerus, M. A., Bibollet-Ruche, F., Telliez, J. B., Wasylyk, B. & Bailleul, B. Serum, AP-1 and Ets-1 stimulate the human ets-1 promoter. *Nucleic Acids Res* **20**, 2699-2703, doi:10.1093/nar/20.11.2699 (1992).

8 Kato, K. *et al.* Structure and functional analysis of the human STAT3 gene promoter: alteration of chromatin structure as a possible mechanism for the upregulation in cisplatin-resistant cells. *Biochim Biophys Acta* **1493**, 91-100, doi:10.1016/s0167-4781(00)00168-8 (2000).

9 Elliott, K. *et al.* Elevated pyrimidine dimer formation at distinct genomic bases underlies promoter mutation hotspots in UV-exposed cancers. *PLoS Genet* **14**, e1007849, doi:10.1371/journal.pgen.1007849 (2018).

10 Fredriksson, N. J. *et al.* Recurrent promoter mutations in melanoma are defined by an extended context-specific mutational signature. *PLoS Genet* **13**, e1006773, doi:10.1371/journal.pgen.1006773 (2017).

11 Perry, R. P. The architecture of mammalian ribosomal protein promoters. *BMC Evol Biol* **5**, 15, doi:10.1186/1471-2148-5-15 (2005).

12 Denisova, E. *et al.* Frequent DPH3 promoter mutations in skin cancers. *Oncotarget* **6**, 35922-35930, doi:10.18632/oncotarget.5771 (2015).

13 Fredriksson, N. J., Ny, L., Nilsson, J. A. & Larsson, E. Systematic analysis of noncoding somatic mutations and gene expression alterations across 14 tumor types. *Nat Genet* **46**, 1258-1263, doi:10.1038/ng.3141 (2014).

14 Nollet, F., Berx, G., Molemans, F. & van Roy, F. Genomic organization of the human beta-catenin gene (CTNNB1). *Genomics* **32**, 413-424, doi:10.1006/geno.1996.0136 (1996).

15 Horn, S. *et al.* TERT promoter mutations in familial and sporadic melanoma. *Science* **339**, 959-961, doi:10.1126/science.1230062 (2013).

16 Huang, F. W. *et al.* Highly recurrent TERT promoter mutations in human melanoma. *Science* **339**, 957-959, doi:10.1126/science.1229259 (2013).

17 Schwachtgen, J. L., Campbell, C. J. & Braddock, M. Full promoter sequence of human early growth response factor-1 (Egr-1): demonstration of a fifth functional serum response element. *DNA Seq* **10**, 429-432, doi:10.3109/10425170009015615 (2000).

18 Falvo, J. V., Tsytsykova, A. V. & Goldfeld, A. E. Transcriptional control of the TNF gene. *Curr Dir Autoimmun* **11**, 27-60, doi:10.1159/000289196 (2010).

19 Ludes-Meyers, J. H. *et al.* Transcriptional activation of the human epidermal growth factor receptor promoter by human p53. *Mol Cell Biol* **16**, 6009-6019, doi:10.1128/MCB.16.11.6009 (1996).
20 Levens, D. How the c-myc promoter works and why it sometimes does not. *J Natl Cancer Inst Monogr*, 41-43, doi:10.1093/jncimonographs/lgn004 (2008).
21 Pfeifer, G. P., Drouin, R., Riggs, A. D. & Holmquist, G. P. Binding of transcription factors creates hot spots for UV photoproducts in vivo. *Mol Cell Biol* **12**, 1798-1804, doi:10.1128/mcb.12.4.1798-1804.1992 (1992).
22 Vorachek, W. R. *et al.* Distant enhancers stimulate the albumin promoter through complex proximal binding sites. *J Biol Chem* **275**, 29031-29041, doi:10.1074/jbc.M003039200 (2000).
23 Gambarotta, G., Pistoi, S., Giordano, S., Comoglio, P. M. & Santoro, C. Structure and inducible regulation of the human MET promoter. *J Biol Chem* **269**, 12852-12857 (1994).
24 Kurukuti, S. *et al.* CTCF binding at the H19 imprinting control region mediates maternally inherited higher-order chromatin conformation to restrict enhancer access to Igf2. *Proc Natl Acad Sci U S A* **103**, 10684-10689, doi:10.1073/pnas.0600326103 (2006).
