# Supplementary material for: Base-resolution UV footprinting by sequencing reveals distinctive damage signatures for DNA-binding proteins
Source: Nat Commun. 2023 May 11;14:2701. doi: 10.1038/s41467-023-38266-2 (PMC10175305; doi:10.1038/s41467-023-38266-2)
Supplement: Supplementary file 3 — Description of Additional Supplementary Files [file 41467_2023_38266_MOESM3_ESM.pdf]

Description of Additional Supplementary Files

File Name: Supplementary Data 1
Description: Data matrix with CPD counts for all dipyrimidine positions in the assayed regions in all samples, plus additional statistics and information.

File Name: Supplementary Software 1
Description: Supplementary zip archive with R and Matlab code written for this study.
