# Supplementary material for: Base-resolution UV footprinting by sequencing reveals distinctive damage signatures for DNA-binding proteins
Source: Nat Commun. 2023 May 11;14:2701. doi: 10.1038/s41467-023-38266-2 (PMC10175305; doi:10.1038/s41467-023-38266-2)
Supplement: Supplementary file 6 — Reporting Summary [file 41467_2023_38266_MOESM6_ESM.pdf]

nature portfolio

Corresponding author(s): Erik Larsson
Last updated by author(s): Mar 13, 2023

Reporting Summary

Nature Portfolio wishes to improve the reproducibility of the work that we publish. This form provides structure for consistency and transparency in reporting. For further information on Nature Portfolio policies, see our Editorial Policies and the Editorial Policy Checklist.

Statistics

For all statistical analyses, confirm that the following items are present in the figure legend, table legend, main text, or Methods section.

n/a	Confirmed	
☐	☒	The exact sample size (*n*) for each experimental group/condition, given as a discrete number and unit of measurement
☐	☒	A statement on whether measurements were taken from distinct samples or whether the same sample was measured repeatedly
☐	☒	The statistical test(s) used AND whether they are one- or two-sided<br>*Only common tests should be described solely by name; describe more complex techniques in the Methods section.*
☐	☒	A description of all covariates tested
☐	☒	A description of any assumptions or corrections, such as tests of normality and adjustment for multiple comparisons
☐	☒	A full description of the statistical parameters including central tendency (e.g. means) or other basic estimates (e.g. regression coefficient) AND variation (e.g. standard deviation) or associated estimates of uncertainty (e.g. confidence intervals)
☐	☒	For null hypothesis testing, the test statistic (e.g. *F*, *t*, *r*) with confidence intervals, effect sizes, degrees of freedom and *P* value noted<br>*Give P values as exact values whenever suitable.*
☒	☐	For Bayesian analysis, information on the choice of priors and Markov chain Monte Carlo settings
☒	☐	For hierarchical and complex designs, identification of the appropriate level for tests and full reporting of outcomes
☒	☐	Estimates of effect sizes (e.g. Cohen's *d*, Pearson's *r*), indicating how they were calculated

Our web collection on statistics for biologists contains articles on many of the points above.

Software and code

Policy information about availability of computer code

Data collection	Capture probe design was done using NimbleDesign, now HyperDesign (https://www.hyperdesign.com; version number not available).
Data analysis	Mapping to hg19 was done using Bowtie 2 version 2.3.1, Picard MergeSamFiles version 2.18.7, and Picard MarkDuplicates version 2.18.7. Conversion from hg38 to hg19 was done using LiftOver version 1.18.0. Data was analysed as outlined in the methods using MATLAB R2022a, R 4.1.0 and Bioconductor 3.14. Scripts are available as a supplementary zip archive.

For manuscripts utilizing custom algorithms or software that are central to the research but not yet described in published literature, software must be made available to editors and reviewers. We strongly encourage code deposition in a community repository (e.g. GitHub). See the Nature Portfolio guidelines for submitting code & software for further information.

Data

Policy information about availability of data

All manuscripts must include a data availability statement. This statement should provide the following information, where applicable:

- Accession codes, unique identifiers, or web links for publicly available datasets
- A description of any restrictions on data availability
- For clinical datasets or third party data, please ensure that the statement adheres to our policy

The Capture CPD-seq data has been deposited in ENA under accession code PRJEB57327 [https://www.ebi.ac.uk/ena/browser/view/PRJEB57327]. A375 naked DNA whole genome CPD-seq generated for this study has been deposited in ENA under accession code PRJEB59730 [https://www.ebi.ac.uk/ena/browser/view/

PRJEB59730]. The following public datasets were used (converted from hg38 to hg19 using LiftOver46 where applicable): A375 whole-genome CPD-seq data in GEO, accession GSE119249 [https://www.ncbi.nlm.nih.gov/geo/query/acc.cgi?acc=GSE119249] . HeLa-S3 RNA-seq from ENCODE, accession ENCFF294GCU [https://www.encodeproject.org/files/ENCFF294GCU/] and ENCFF496CRF [https://www.encodeproject.org/files/ENCFF496CRF/]. HeLa-S3 DNase I peaks [https://hgdownload.cse.ucsc.edu/goldenPath/hg19/encodeDCC/wgEncodeAwgDnaseUniform/wgEncodeAwgDnaseUwdukeHelas3UniPk.narrowPeak.gz].
Hela-S3 NFY-A ChIP peaks [https://hgdownload.cse.ucsc.edu/goldenPath/hg19/encodeDCC/wgEncodeAwgTfbsUniform/wgEncodeAwgTfbsSydhHelas3NfyaIggrabUniPk.narrowPeak.gz].
Hela-S3 NFY-B ChIP peaks [https://hgdownload.cse.ucsc.edu/goldenPath/hg19/encodeDCC/wgEncodeAwgTfbsUniform/wgEncodeAwgTfbsSydhHelas3NfybIggrabUniPk.narrowPeak.gz].
Hela-S3 ELK 1 ChIP peaks [https://hgdownload.cse.ucsc.edu/goldenPath/hg19/encodeDCC/wgEncodeAwgTfbsUniform/wgEncodeAwgTfbsSydhHelas3Elk112771IggrabUniPk.narrowPeak.gz].
Hela-S3 ELK 4 ChIP peaks [https://hgdownload.cse.ucsc.edu/goldenPath/hg19/encodeDCC/wgEncodeAwgTfbsUniform/wgEncodeAwgTfbsSydhHelas3Elk4UcdUniPk.narrowPeak.gz].
GM12878 SRF ChIP peaks [https://hgdownload.cse.ucsc.edu/goldenPath/hg19/encodeDCC/wgEncodeAwgTfbsUniform/wgEncodeAwgTfbsHaibGm12878SrfPcr2xUniPk.narrowPeak.gz].
HeLa-Se DNase I footprints, HeLa-S3-DS24790 [https://resources.altius.org/~jvierstra/projects/footprinting.2020/per.dataset/HeLa_S3-DS24790/]. Foreskin melanocyte DNase I data from Roadmap Epigenomics, accession number E059 [https://egg2.wustl.edu/roadmap/data/byFileType/peaks/consolidated/narrowPeak/E059-DNase.hotspot.all.peaks.v2.bed.gz]. Factorbook ETS (ETS1, ELK4, GABPA, ELF1), SRF and NF-Y binding sites were obtained from UCSC (http://hgdownload.soe.ucsc.edu/goldenPath/hg19/database/factorbookMotifPos.txt.gz). ENCODE clustered transcription factor binding sites were obtained from UCSC [http://hgdownload.soe.ucsc.edu/goldenPath/hg38/encRegTfbsClustered/encRegTfbsClusteredWithCells.hg38.bed.gz]. Cancer Cell Line Encyclopedia HeLa copy number data was downloaded from DepMap (20Q2 release) [https://doi.org/10.6084/m9.figshare.12280541.v4]. WGS-based melanoma mutation calls from the Australian Melanoma Genome Project were attained via the ICGC database [https://dcc.icgc.org/projects/MELA-AU]. TCGA WGS melanoma mutation calls were based on alignments downloaded from cgHub (cgHub is no longer available; TCGA WGS data is now accessible through Genomic Data Commons [https://portal.gdc.cancer.gov]). Researchers need to apply for access to TCGA WGS data to the TCGA Data Access Committee (DAC) via dbGaP (https://dbgap.ncbi.nlm.nih.gov). Source data are provided with this paper.

Human research participants

Policy information about studies involving human research participants and Sex and Gender in Research.

Reporting on sex and gender	Not relevant
Population characteristics	Not relevant
Recruitment	Not relevant
Ethics oversight	Not relevant

Note that full information on the approval of the study protocol must also be provided in the manuscript.

Field-specific reporting

Please select the one below that is the best fit for your research. If you are not sure, read the appropriate sections before making your selection.

☒ Life sciences ☐ Behavioural & social sciences ☐ Ecological, evolutionary & environmental sciences

For a reference copy of the document with all sections, see nature.com/documents/nr-reporting-summary-flat.pdf

Life sciences study design

All studies must disclose on these points even when the disclosure is negative.

Sample size	n=3 for each cellular condition (untreated, stimulated, starved) and n=9 naked replicates. In cell-to-naked comparisons, cellular samples were combined (n=9) and compared to the naked DNA replicates. No sample size calculation was done: the number of cellular (n=9) and naked (n=9) samples was chosen as a reasonable balance between statistical power and practical feasibility.
Data exclusions	For "No UV" samples three untreated cellular samples were used, while six additional "no UV" samples were generated but not considered in the study.
Replication	No separate replication studies have been performed. The genomic nature of the data allowed replicate observations of damage signatures for specific proteins to be obtained through investigation of different genomic sites (e.g. Fig. 4), which supported the general conclusions.
Randomization	Randomization is not relevant as this is an in vitro study based on the HeLa cell line.
Blinding	No blinding was done. It is not possible to perform these experiments in a blinded fashion.

Reporting for specific materials, systems and methods

We require information from authors about some types of materials, experimental systems and methods used in many studies. Here, indicate whether each material, system or method listed is relevant to your study. If you are not sure if a list item applies to your research, read the appropriate section before selecting a response.

Materials & experimental systems

n/a	Involved in the study
☒	☐ Antibodies
☐	☒ Eukaryotic cell lines
☒	☐ Palaeontology and archaeology
☒	☐ Animals and other organisms
☒	☐ Clinical data
☒	☐ Dual use research of concern

Methods

n/a	Involved in the study
☒	☐ ChIP-seq
☒	☐ Flow cytometry
☒	☐ MRI-based neuroimaging

Eukaryotic cell lines

Policy information about cell lines and Sex and Gender in Research

Cell line source(s)	HeLa cells from CLS cell lines service 300194 (https://cls.shop/HeLa/300194)
Authentication	Not authenticated
Mycoplasma contamination	Negative mycoplasma
Commonly misidentified lines (See ICLAC register)	No commonly misidentified cell lines were used
